# Winning Isn't Everything: Mood and Testosterone Regulate the Cortisol Response in Competition

**DOI:** 10.1371/journal.pone.0052582

**Published:** 2013-01-09

**Authors:** Samuele Zilioli, Neil V. Watson

**Affiliations:** Behavioral Endocrinology Laboratory, Department of Psychology, Simon Fraser University, Burnaby, Canada; University of Washington, United States of America

## Abstract

Dominance contests are recurrent and widespread causes of stress among mammals. Studies of activation of the stress axis in social defeat – as reflected in levels of adrenal glucocorticoid, cortisol – have generated scattered and sometimes contradictory results, suggesting that biopsychological individual differences might play an important mediating role, at least in humans. In the context of a larger study of the regulation of endocrine responses to competition, we evaluated the notion that mood states, such as self-assurance and hostility, may influence cortisol reactivity to dominance cues via an interplay with baseline testosterone, considered as a potential marker of individual differences in dominance. Seventy healthy male university students (mean age 20.02, range 18–26) provided saliva samples before and after competing for fifteen minutes on a rigged computer task. After a winner was determined, all participants were assessed on their mood states through a standardized psychometric instrument (PANAS-X). Among winners of a rigged videogame competition, we found a significant interaction between testosterone and self-assurance in relation to post-competition cortisol. Specifically, self-assurance was associated with lower post-competition cortisol in subjects with high baseline testosterone levels, but no such relationship was observed in subjects with lower baseline testosterone levels. In losers of the competition no interaction effect between basal testosterone and hostility was observed. However, in this subgroup a significant negative relationship between basal testosterone and post-competition cortisol was evident. Overall, these findings provide initial support for the novel hypothesis that biological motivational predispositions (i.e. basal testosterone) and state (i.e. mood changes) may interact in regulating the hypothalamic-pituitary-adrenal axis activation after a social contest.

## Introduction

Dominance contests are ubiquitous, recurrent causes of stress among mammalian species. The end product of the hypothalamic-pituitary-adrenal (HPA) axis, cortisol, is widely used as a physiological proxy for this stress response [1]. Early research in the field seemed to suggest that animals that are social subordinates, as established in agonistic encounters, would show a stronger adrenocortical response compared to victorious conspecifics [2], [3]. These findings were confirmed by subsequent and more recent studies in both naturalistic [4] and captive settings [5], [6].

In humans, empirical evidence in favor of a greater activation of the stress axis in response to social defeat is more equivocal (for a review see [7]) – one possibility is that mediation by biopsychological individual differences might account for this observed variability in responses. Motivation to gain status might be one such mediating characteristic [8], [9]. Wirth and collaborators [8] found that the intensity of the individual's intrinsic need to enhance their own status relative to others, termed “implicit power motivation”, was a crucial moderator of the effect of competitive outcome on cortisol responses. Specifically, “high power” individuals showed a decrease in cortisol after victory and an increase in the same hormone after losing. An opposite pattern was found in “low power” subjects, for whom victory was more stressful than defeat. Because of the link between baseline testosterone and status-seeking behavior [10] and testosterone's correlation with implicit power motivation [11], Mehta and colleagues [9] proposed basal testosterone as an additional regulator of the HPA reactivity to social victory and defeat. Results of their experiments showed that only in high testosterone individuals was cortisol affected by the outcome of the competition. In fact, while low testosterone participants did not experience any change in cortisol after the competition, supposedly because of the absence of preference for status in this subgroup, high testosterone individuals experienced a decrease in cortisol after winning, but an increase in cortisol after losing. These physiological responses appeared to regulate subsequent behaviors, such that only high testosterone winners decided to repeat the competition, highlighting the importance of including measures of baseline testosterone in further investigation of HPA activation in dominance contests.

Another factor that may modulate hormonal responses to social confrontation is mood (for a review, see [12]). In their model of neuroendocrine and mood responses to a competitive situation, Salvador and Costa [12] identified mood changes as products of individual coping mechanisms and proposed a negative correlation between changes in cortisol and changes in mood. Two scenarios were identified. In the first one, it was proposed that positive changes in mood after competition, originating from active patterns of coping (e.g., appraising the situation as controllable), would be associated with more sympathetic adrenomedullary (SAM) response to stress than cortisol stress response. On the other hand, passive patterns of coping (e.g., appraising the situation as uncontrollable) would be associated with adrenocorticotropic-releasing hormone (ACTH) release and consequent elevation of cortisol. Thus, these authors posit a negative correlation between mood and cortisol (with reduced mood related to increase in cortisol secretion); and while the direction of causality is not specified, they leave open the possibility of a direct influence of mood fluctuations on cortisol output, as suggested in other studies (e.g., [13]).

The present study extends the notion that mood changes may influence cortisol reactivity to dominance cues, by examining their relationship to basal testosterone, employed here as a biological indicator of individual differences in dominance [14]. Thus, affect may be a significant predictor of cortisol changes only in those subjects that would be expected to be biologically more oriented towards achieving and maintaining high status: namely, high testosterone individuals. While it is certainly true that variation in testosterone is only one aspect of the more complex concept of dominance, the large literature linking naturally occurring and experimentally elevated concentrations of this steroid with dominance makes the hypothesis worthy of investigation [10]. In addition, it seems plausible that specific aspects of affect might be selectively involved, rather than overall, general positive or negative mood. For example, previous research suggests that hostility exacerbates cardiac [15], [16], endocrine [17], [18], and reduces immune [19], [20], [21] responses to stress. Suarez and collaborators [18] found that high-hostility subjects, who were harassed during a solvable task, showed stronger cortisol and norepinephrine reactivity compared to low-hostility subjects. Likewise, Pope and Smith [17] found that men that scored high on hostility exhibited significantly greater adrenocortical excretion during typical daily activities than did low-hostility men. Consequently, we reasoned that hostility and its interaction with basal testosterone might play a role in stress responses to social defeat. Under this model, therefore, higher-hostility high-basal testosterone losers would have more prominent cortisol responses than lower- hostility high-basal testosterone losers. In other words, social defeat should cause a rise in cortisol in “dominant” people (inferred from testosterone levels, subject to the limitations discussed earlier) only if they experienced high transient feelings of hostility following the loss.

As we have noted, winning or losing are not, by themselves, good predictors of HPA stress axis activation (see for example, [9]). In other words, winners of social contests are not less immune to cortisol reactivity than losers. One possible moderator of the HPA response after social victory may be positive affect: more specifically, self-assurance [22]. A parallel can be drawn between this mood state and personality traits reportedly linked to cortisol responsiveness, such as self-confidence (see for example, [23] and [24]) and mental toughness [25]. For instance, Salvador and colleagues [23] found that self-confidence correlated with pre-competition cortisol. But when Flegr and Priplatova [24] measured hormonal parameters *during* and *after* a university written exam, self-confidence was found to have a negative relationship with cortisol. Based on this pattern of results, we reasoned that winners experiencing states of reduced confidence would have a more marked adrenocortical response than that occurring in more confident winners. So for example, a winner that feels proud, strong and confident [22] about his victory or aspects of it would undergo a decrease in cortisol compared to a winner that does not enjoy such high levels of self-assurance. This pattern may be especially evident for people with high basal levels of testosterone compared to subjects with less biological predisposition to dominance.

The Positive and Negative Affect Schedule – Expanded (PANAS-X [22]) allows measurement of these specific mood states (hostility and self-assurance) while controlling for other emotional states. In this scale subjects use emotional adjectives to describe their current mood, which is profiled in terms of multiple specific individual affective states: fear, sadness, guilt, hostility, shyness, fatigue, surprise, joviality, self-assurance, attentiveness, and serenity. Higher-order composite scales measuring “positive” and “negative” mood can also be calculated. Hence, we used this standardized psychometric instrument to study how self-assurance and hostility *states* (not *traits*, as conceptualized in previous studies; [22]) may interact with pre-competition basal testosterone to moderate cortisol release in losers and winners of a rigged competition. To our knowledge, this is the first study to examine how specific positive and negative affect states, acting jointly with basal testosterone, may influence HPA activation to social stress.

## Methods

### Participants

Seventy male undergraduates (mean age = 20.3 years, SD = 2.31, range = 8) participated in exchange for course credit in an introductory psychology class, in the context of a larger study of hormone responses in competition. An analysis of endocrine regulation of testosterone responses deriving from this dataset appears in Zilioli and Watson [26], along with additional procedural details.

To minimize inter-individual variation in hormone levels due to sex [27] only males were recruited for this study. Participants were screened before the beginning of the experiment and excluded if they reported neuroendocrine dysfunctions, regular use of recreational drugs, and/or chronic or recent intake of prescription medications known to influence hormonal levels. All procedures were subject to review and prior approval by the Simon Fraser University Research Ethics Board.

### Procedure

All testing sessions were scheduled between 1400 h and 1900 h, to control for the effects of diurnal variation in testosterone and cortisol secretion [28], [29], [30], [31]. Upon arriving, each participant was directed by a male experimenter to one of two small rooms, where they completed an informed-consent form and a simple questionnaire sampling bio-demographic information (e.g., height, weight, sexual orientation, and educational level). Subjects also immediately provided a baseline saliva sample (time one; T1). During this period participants were given instructions for the competition task and informed that the winner would receive a $10 cash prize. After providing the first saliva sample, participants underwent the experimental manipulation, which consisted in a rigged competition on a modified version of a well-known commercial videogame, Tetris (for details, see [26]). Following the competition participants completed, in order, the mood and attribution measures, described below, and viewed a neutral video (a documentary about Ireland, serving as a filler task). At exactly 30 minutes after the completion of the Tetris competition, participants provided a second saliva sample (time two; T2) [11], [32], [33], [34] and were given a printed debriefing form to read.

### Paper-and-pencil measures

Mood. Immediately following the competition task, subjects completed the PANAS-X [22], which is a self-rating scale designed to assess a variety of mood states. It was validated on the basis of the Profile of Mood States (POMS; [35]); however, its stronger discriminant validity and the ease of administration make it a better alternative to POMS. PANAS-X scales are usually completed in about 5 minutes. During the questionnaire, subjects are asked to rate 60 adjectives on a scale from one (not at all) to five (extremely) to indicate their mood at the moment of its administration. Each scale is the result of the combination (i.e. average) of some of these adjectives. For example, the scale “Fear” contains the following adjectives: afraid, scared, frightened, nervous, jittery and shaky.

#### Attribution survey

In order to examine participants' attributions for the competition outcome, we created an *ad hoc* survey using 5-point Likert-type questions assessing the role of personal ability and luck, as well as open questions (e.g., *“Why do you think you have lost?”*) [36], [37]. The attribution survey was also designed to: (1) check for suspicions about the rigged nature of the contest; (2) provide general feedback from participants about the competition and the experiment up to that point; and, (3) explore whether the experimental manipulation had an impact on other psychological processes, specifically perceived control over the competition outcome (e.g.,*“How much control did you have over whether you won or lost”*) [38].

### Saliva samples and hormone assays

Participants were instructed to abstain from eating, drinking, smoking, or brushing their teeth for one hour before testing. Saliva samples were collected using oral swabs (Salimetrics LLC, State College, PA) placed under the tongue. Samples were chilled immediately following collection, and then frozen within one hour and held at −20°C until assay. On the day of the assay, frozen samples were first warmed to room temperature and then centrifuged (3000 rpm) for 15 minutes in order to extract saliva from the swabs. Samples were then assayed in duplicate using competitive enzyme immunoassays for testosterone and cortisol (Salimetrics). The average intra-assay coefficient of variation was below 5% for both testosterone and cortisol, and inter-assay coefficients for low and high control were 13.4% and 9.3% for testosterone, and 3.1% and 8.2% for cortisol. Steroid levels at baseline were in the normal ranges (testosterone: M = 113.7 pg/mL, SD = 44.1, cortisol: M = .15 µg/dL, SD = .09) (Salimetrics).

### Statistical analyses

In order to assess whether there were any differences between winners and losers on socio-demographic variables or hormonal levels before the competition, we performed several independent-groups t-tests. Pearson product-moment correlations were used to assess associations between continuous variables. The effect of competition on cortisol was tested via repeated-measures ANOVA. Keeping winners and losers separate, variables of interest were centered by subtracting the sample mean on a specific variable from each subject's score on that variable. Linear multiple regression analyses (see Results section for details) were then carried out to investigate the interaction of basal testosterone and mood on changes in cortisol. Simple-slope analyses were used to interpret potential interaction effects [39], [40]. And lastly, assumptions were checked for each linear regression via analyses of the residuals (i.e., the form of the relation between the dependent variable and the independent variables, normality of the residuals, constant variance of residuals). All tests are two-tailed (α = .05) and were carried out using PASW (SPSS) Statistics 17.0.3.

## Results

Participants (n = 10) who reported suspicion about the nature of the competition were removed from the analysis, leaving a final sample of 60 participants (30 losers).

On the basis of Box-plot inspection, which makes no assumption about the data distribution [41], outliers were identified in both baseline cortisol (n = 4, two losers) and baseline testosterone (n = 1, one loser). In the case of cortisol outliers, two subjects were nearly three standard deviations from the sample mean, one subject was 3.4 standard deviations from the mean, and the remaining outlier was 6.5 standard deviations from the mean. In the case of testosterone, the outlier was 4.4 standard deviations from the mean. These individuals were excluded from further statistical analyses.

### Preliminary analyses

#### Competition Outcome

The randomly assigned “winners” and “losers” did not differ on any bio-demographic variables (age, height, weight, education) [t-test, *ns*]. They also did not differ with regard to past involvement with videogaming, physique (BMI), or preceding night's sleep. Independent-groups *t*-tests further confirmed that at baseline, winners and losers did not differ in their salivary concentrations of testosterone [*t* (53) = −0.089, *ns*] or cortisol [*t* (53) = −0.428, *ns*]. The experimental manipulation was effective in causing differences between winners and losers in both negative affect [*t* (49.919) = −2.871, *p*<0.01) and positive affect (*t* (53) = 3.068, *p*<0.01]. In line with these findings, winners scored higher on all three basic positive emotion scales [attentiveness, *t* (53) = 2.095, *p*<0.05; joviality, *t* (53) = 5.121, *p*<0.001; and self-assurance, *t* (53) = 2.685, *p*<0.05] whereas, losers reported higher scores on three of the four basic negative emotion scales [hostility, *t* (53) = −2.738, *p*<0.01; sadness, *t* (53) = −3.474, *p*<0.001; and guilt, *t* (35.818) = −4.657, *p*<0.001]. Of the other affective states measured by the PANAS-X only surprise showed a significant difference, with winners scoring higher [*t* (53) = 2.442, *p*<0.05].

#### Hormones and Potential Confounds

Table 1 presents correlations between baseline hormone concentrations and variables identified as potential nuisance factors in previous research (e.g., [42]). These factors include age, BMI, and collection time (hour of the day saliva sample was gathered). A significant negative correlation was observed between baseline cortisol and collection time [*r* = −0.276, *p*<0.05] as well as between baseline cortisol and changes in cortisol [*r* = −.64, *p*<0.001]. A significant positive correlation was found between basal testosterone and age [*r* = 0.276, *p*<0.05]. Additionally, consistent with previous studies [9], [42] baseline cortisol positively correlated with baseline testosterone [*r* = 0.53, *p*<0.001]. Detailed descriptive statistics are reported in Table 2.

**Table 1:** Correlations among hormonal measures and potential confounders (n = 55).

	Baseline T	Baseline C	Δ C	Δ T	Age	Time	BMI
Baseline T		.53**	−.104	−448**	.276*	−.209	−.056
Baseline C			−.65**	−.53**	.172	−.276*	−.065
Δ C				.315*	−.052	.044	−.053
Δ T						.107	.101
Age						.044	.232
Time							.06

* *p*<0.05,

** ≤0.001.

**Table 2:** Descriptive statistics for key demographic variables in winners (n = 28) and losers (n = 27).

	WINNERS		LOSERS	
	M (SEM)	SD	M (SEM)	SD
Pre-competition testosterone (pg/mL)	110.12 (8.37)	44.3	111.17 (8.3)	43.18
Pre-competition cortisol	0.13 (0.01)	0.07	0.14 (0.02)	0.1
Age	20.32 (0.44)	2.34	19.7 (0.43)	2.23
Collection time	17:32 (00:14)	01:14	17:16 (00:15)	01:20
BMI	22.54 (0.62)	3.28	24.07 (0.85)	4.44
Self-Assurance (PANAS X)	3.14 (0.16)	0.85	2.57 (0.14)	0.7
Hostility (PANAS X)	1.4 (0.1)	0.5	1.8 (0.1)	0.59

#### Hormones and Mood

We hypothesized that the pattern of association between components of affect and baseline testosterone in predicting post-competition changes in cortisol may differ between winners and losers. Accordingly, correlation matrices for baseline testosterone, baseline cortisol, the PANAS-X scales and individual measures of perceived control are presented separately for winners and losers (see Table 1 and Table 2 in Appendix S1).

### Cortisol and Testosterone Pre- and Post-Competition

The effect of the competition manipulation on cortisol secretion was assessed via repeated measures 2×2 factorial ANOVA, with outcome (victory or defeat) as a between-subjects factor and time (pre-competition (T1) cortisol vs. post-competition (T2) cortisol) as a within-subjects factor. Results revealed a significant main effect of time on cortisol, *F* (1,53) = 12.704, *p*<.01, but no significant interaction between time and outcome on cortisol, *F* (1,53) = 1.797, *ns*. Overall cortisol declined from baseline over the course of the test session, and it did so equivalently in winners and losers. When looking at percentage change, winners had an average cortisol decrease of 7% (SE 6.7) and an average testosterone increase of 3.4% (SE 3.1); on the other hand, losers had an average cortisol decrease of 9% (SE 12.3) and an average testosterone decrease of 7% (SE 2.7).

### Interaction between baseline testosterone and hostility in relation to post-defeat Cortisol

To determine if basal testosterone and hostility interact in predicting changes in cortisol a hierarchical regression analysis was run on the competition losers. Specifically, post-competition cortisol was entered as dependent variable and the following variables as predictors: collection time, age and basal cortisol in Step 1; hostility and basal testosterone in Step 2; the interaction between basal testosterone and hostility in Step 3. This analysis indicated that all three linear regression models were significant, but adding the interaction between basal testosterone and hostility in Step 3 did not significantly increase the amount of variance explained in predicting post-competition cortisol [Δ *F* (1,20) = .027, *ns*, Δ *R* ^2^ = .001]. However, when adding basal testosterone and hostility (Step 2) we observed an increase in the amount of variance explained [Δ *F* (2,21) = 4.069, *p*<.05, Δ *R* ^2^ = .167]. Of the two new variables included in the model [*R* ^2^ = .571, adjusted *R* ^2^ = .442, *F* (5,21) = 5.571, *p*<.01] only a main effect of basal testosterone was found [b = .416, t (21) = 2.434, p<.05], with no significant contribution of hostility [b = −.221, t (21) = −1.502, *ns*]. Overall, this analysis indicated that pre-competition baseline testosterone moderated the effects of defeat on cortisol changes: Increased cortisol post-contest was associated with high levels of basal testosterone among losers regardless of their hostility.

### Interaction between baseline Testosterone and Self-Assurance in relation to post-victory Cortisol

To examine if basal testosterone and self-assurance interact in predicting changes in cortisol a hierarchical regression analysis was run on the winners group. Specifically, post-competition cortisol was entered as dependent variable and the following variables as predictors: collection time, age and basal cortisol in Step 1; self-assurance and basal testosterone in Step 2; the interaction between basal testosterone and self-assurance in Step 3. This analysis indicated that all three linear regression models were significant; however, when adding basal testosterone and self-assurance (Step 2) we did not observe a significant increase in the amount of variance explained [Δ *F* (2,22) = 2.876, *ns*, Δ *R* ^2^ = 0.48], while adding the interaction between basal testosterone and self-assurance in Step 3 increased the amount of variance explained in predicting post-competition cortisol [Δ *F* (1,21) = 4.626, *p*<.05, Δ *R* ^2^ = .033]. The statistics for the final model were: *R* ^2^ = .850, adjusted *R* ^2^ = .808, *F* (6,21) = 19.907, *p*<.001.

**Figure 1:**
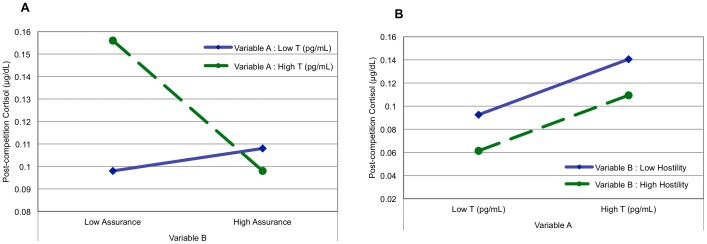
Relationship between testosterone, mood and cortisol reactivity after competition. **A**. Post-competition cortisol (µg/dL) in winners (n = 28) as a function of Variable A (basal testosterone -pg/mL-) and Variable B (self-assurance levels) after controlling for age and collection time. Low = 1 standard deviation below mean; high = 1 standard deviation above mean. When pre-competition T was high, self-assurance was related to post-competition cortisol after victory, with higher increase in those participants that reported less self-assurance. **B**. Post-competition cortisol (µg/dL) in losers (n = 27) as a function of Variable A (basal testosterone -pg/mL-) and Variable B (hostility levels) after controlling for age and collection time. Low = 1 standard deviation below mean; high = 1 standard deviation above mean. The parallel lines indicate absence of interaction between hostility and basal testosterone; however, regardless the mood, losers with high T experienced a significant increase in cortisol in respect to losers with low basal T.

To interpret the significant interaction, we first conducted a simple slope analysis for basal testosterone 1 SD below the mean and 1 SD above the mean [39], [40]. Subsequently, we graphed the interaction by plotting post-competition cortisol 1 SD above and 1 SD below the means for basal testosterone and self-assurance (Figure 1A). For baseline testosterone 1 SD below the mean, the slope did not significantly differ from zero [b = .061, *t* (21) = .407, *ns*]. In contrast, a significant effect was found for baseline testosterone 1 SD above the mean [b = −.418, *t* (21) = −3.001, *p*<0.01], reflecting a significant negative association between self-assurance and cortisol changes at high levels of basal testosterone. Taken together, these data indicate that for individuals with higher pre-competition testosterone, – but not for lower baseline testosterone individuals – self-assurance induced by competition predicted changes in cortisol after victory. Specifically, those people with high basal testosterone who felt less confident and strong after the contest experienced a larger increase in cortisol following victory. Figure 1B shows the *non*-significant interaction between hostility and basal testosterone in competition losers.

## Discussion

Our hypothesis of a negative association between self-assurance and cortisol output in high-testosterone winners was confirmed, indicating that cortisol responses to stressful events (i.e. competition) are not independent of basal testosterone (a partial biological proxy for dominance) and some mood state (i.e. self-assurance) (Figure 1).

A possible explanation for the association between self-assurance and cortisol responses to stress could be that the level of confidence experienced after the contest reflects the appraisal of the event, with less positive affect resulting in an evaluation of the event as more challenging and threatening [43]. Likewise, lower self-assurance might indirectly reflect a more passive and less effective coping mechanism, which is associated with ACTH and cortisol secretion rather than sympathetic adrenomedullary activation and release of adrenalin [44]. This hypothesis has been confirmed not only in humans (for a review, see [12]), but also in a variety of other species (for a review, see [45]). Furthermore, a more passive coping style could be possibly associated with slower recovery period, which translates into an enduring effect of the stressor over a longer time in those individuals with less functional coping ability.

The negative association between self-assurance and reactive cortisol in high-testosterone winners also dovetails with both empirical [24] and theoretical [25] evidence linking HPA activity and constructs related to self-assurance (i.e. self-confidence and mental toughness). Further supporting these parallels we found a positive correlation trend (*r* = .356, *p* = .063) between pre-competition cortisol and self-assurance in winners (see Table 1 in Appendix S1), as previously reported in competition studies looking at the relationship between self-confidence and hormones [23], [46]. It is important to note however, that our correlation between pre-competition hormone status and post-competition mood measures differs from previous studies where self-confidence was measured *before* the contest onset. This similarity makes sense though if we define state affect (i.e. transient fluctuation in mood) as short-term deviation from the responder-typical (mean) mood (i.e. trait affect) [47]. Of course, state affect defined in this manner encompasses not only transient aspects of mood, but also individual trait variability. Our data agree with broader findings linking positive mood (state *and* trait) to lower HPA activity [48], [49], [50].

Lastly, the moderating role of self-assurance was evident only in high-testosterone men – low-testosterone individuals showing no cortisol response – consistent with the idea that basal testosterone partly taps into the emotional-motivational disposition towards dominance (i.e. achieving and maintaining high status) [10], [14]. This relationship between self-assurance and testosterone seems to be relevant to physiological [9], [51] and behavioral responses [9] to varying social situations, such as competition outcomes [52]. In other words, the moderating effect of mood on cortisol may manifest itself only in individuals with a high drive for status (namely, high-testosterone subjects), with low-testosterone individuals being less affected by mood changes considering their almost absent physiological responses when dealing with status shift [9], [51], [53]. For example, Mehta and colleagues [9] found that the competition outcome had no significant effect on the cortisol response of men with low-testosterone. In addition, when cortisol changes were regressed on basal testosterone, only the positive correlation between testosterone and cortisol changes in losers was found to be significant, whereas the negative correlation between the same variables in winners was found not to be significant. These data seem to suggest that additional individual differences (e.g., mood states and coping style) may interact with basal testosterone in predicting cortisol changes in the winner condition, and that is exactly what we found in our study.

Mehta et al.'s empirical data [9] are also consistent with what we found in the loser condition: Basal testosterone by itself served as a good predictor of cortisol changes in these subjects (Figure 1B). In accordance with Mehta et al.'s finding, men with initial high testosterone concentrations showed an increase in cortisol after losing the competition. As suggested by those authors there are two possible explanations that could account for such interaction. First, in accordance with previous studies investigating HPA activity and dominance in humans [8] and mice [6], it is possible that heightened cortisol after social defeat serves as an indicator of social stress especially for those individuals with a stronger motivation to gain high status. Additionally, this physiological response may be functional, acting to liberate energy (via mobilization of glucose) needed for further efforts, to regain status. In this case, dynamic fluctuation in cortisol after defeat might be a marker of motivational state as shown in recent reports (e.g., [54]).

The suggested interplay between hostility and basal testosterone in predicting adrenocortical reactivity in losers was not confirmed. One possibility is that the PANAS-X was not sensitive enough to detect the hypothesized effect. Alternatively, trait hostility may be a more reliable predictor of changes cortisol than is state hostility (e.g., [18]). Of course, it may also be the case that population levels of testosterone-affect interaction play little role in determining cortisol in competition losers. This issue remains to be determined in future research utilizing alternative measures of mood state (i.e., [13]) in larger or alternative samples. Future studies on this topic would also benefit from including an estimate of individual differences in stress reactivity, an important variable that we did not control in the current report. For example, the magnitude of the response of the HPA axis could be assessed a few days prior the experiment by employing a standardized acute laboratory paradigm (for example the Trier Social Stress Test (TSST); [55]. This information could be used later to distinguish responder and non-responder (see for example, [56]), an important covariate (or additional factor) in the experimental design. Additionally, as we had no *a priori* hypotheses concerning race/ethnicity and assume that any associated error variance would distribute randomly across experimental conditions, we did not collect this information from most of our subjects. Future studies may wish to address this question. And lastly, future studies could investigate the same phenomenon in women, shedding light on the complex relationship between biological sex, social environment and hormonal manifestations.

### Conclusions

The aim of the current study was to investigate the interaction between basal testosterone and specific mood states in predicting cortisol changes after a social dominance contest, where the competition outcome was randomly assigned. For winners, we found a significant interaction between pre-competition testosterone and self-assurance in relation to post-competition cortisol, such that high self-assurance was associated with low post-competition cortisol, but only in subjects with high pre-competition testosterone levels. No such relationship was evident in subjects with low pre-competition testosterone levels (Figure 1A).

For losers, although no interaction effect was observed between pre-competition testosterone and hostility with respect to post-competition cortisol, there was a significant overall negative relationship between baseline testosterone and post-competition cortisol.

Taken together, these findings support the emerging view that some biological motivational predispositions (i.e. basal testosterone/dominance) and state (i.e. mood changes) interact in regulating activation of the hypothalamic-pituitary-adrenal stress axis after a social contest.

## Supporting Information

Appendix S1 **Correlations among hormonal measures, perceived control and mood.**
(DOCX)
