# Supplementary material for: Winning Isn't Everything: Mood and Testosterone Regulate the Cortisol Response in Competition
Source: PLoS One. 2013 Jan 9;8(1):e52582. doi: 10.1371/journal.pone.0052582 (PMC3541278; doi:10.1371/journal.pone.0052582)
Supplement: Appendix S1 — Correlations among hormonal measures, perceived control and mood. (DOCX) [file pone.0052582.s001.docx]

**Appendix S1**

Table 1. Correlations among hormonal measures, perceived control and mood in winners (n=28). **p* ≤0.01, ***p* ≤0.001

|  | I | II | III | IV | V | VI | VII | VIII | IX | X | XI | XII | XIII | XIV | XV | XVI |
| --- | --- | --- | --- | --- | --- | --- | --- | --- | --- | --- | --- | --- | --- | --- | --- | --- |
| I. Baseline T | 1.00 |  |  |  |  |  |  |  |  |  |  |  |  |  |  |  |
| II. Baseline C | .551^**^ | 1.00 |  |  |  |  |  |  |  |  |  |  |  |  |  |  |
| III. Negative aff. | .359 | .413 | 1.00 |  |  |  |  |  |  |  |  |  |  |  |  |  |
| IV. Positive aff. | -.030 | .340 | .197 | 1.00 |  |  |  |  |  |  |  |  |  |  |  |  |
| V. Attentiveness | -.173 | .212 | .085 | .907^*^ | 1.00 |  |  |  |  |  |  |  |  |  |  |  |
| VI. Shyness | .073 | .082 | .600^**^ | -012. | -087 | 1.00 |  |  |  |  |  |  |  |  |  |  |
| VII. Fatigue | -.185 | -.25 | .148 | -.484^*^ | -.476^*^ | .517^*^ | 1.00 |  |  |  |  |  |  |  |  |  |
| VIII. Serenity | -.245 | -.421 | -.435 | -.419 | -.302 | -.1 | .397 | 1.00 |  |  |  |  |  |  |  |  |
| IX. Surprise | -.223 | .277 | .083 | .485^*^ | .47 | .027 | -.242 | -.528^*^ | 1.00 |  |  |  |  |  |  |  |
| X. Fear | .210 | .345 | .753^**^ | .426 | .268 | .342 | .016 | -.417 | .326 | 1.00 |  |  |  |  |  |  |
| XI. Hostility | .293 | .28 | .682^**^ | .114 | .013 | .662^**^ | .172 | -.227 | -.103 | .229 | 1.00 |  |  |  |  |  |
| XII. Guilt | .22 | .185 | .613^**^ | -.05 | -.156 | .531^*^ | .505^*^ | .022 | -.219 | .475 | .497^*^ | 1.00 |  |  |  |  |
| XIII. Sadness | .16 | .051 | .445 | -.084 | -.211 | .601^**^ | .504^*^ | .187 | -.232 | .303 | .417 | .829^**^ | 1.00 |  |  |  |
| XIV. Joviality | .049 | .409 | .203 | .893 | .761^**^ | .025 | -.46^*^ | -.520^*^ | .572^**^ | .469 | .082 | .006 | -.027 | 1.00 |  |  |
| XV. Assurance | .052 | .356 | .201 | .904^**^ | .727 | -.059 | -.43 | -.446 | .512^*^ | .442 | .05 | -.037 | -.041 | .791^**^ | 1.00 |  |
| XVI. Control | -.03 | -.074 | -.279 | .385 | .373 | -.26 | -.288 | .195 | -.206 | -.065 | -.074 | -.152 | -.164 | .246 | .301 | 1.00 |

Table 2. Correlations among hormonal measures, perceived control and mood in losers (n=27). **p* ≤0.01, ***p* ≤0.001

|  | I | II | III | IV | V | VI | VII | VIII | IX | X | XI | XII | XIII | XIV | XV | XVI |
| --- | --- | --- | --- | --- | --- | --- | --- | --- | --- | --- | --- | --- | --- | --- | --- | --- |
| I. Baseline T | 1.00 |  |  |  |  |  |  |  |  |  |  |  |  |  |  |  |
| II. Baseline C | .523^*^ | 1.00 |  |  |  |  |  |  |  |  |  |  |  |  |  |  |
| III. Negative aff. | .041 | -.01 | 1.00 |  |  |  |  |  |  |  |  |  |  |  |  |  |
| IV. Positive aff. | -.057 | .072 | .314 | 1.00 |  |  |  |  |  |  |  |  |  |  |  |  |
| V. Attentiveness | .031 | .156 | .284 | .856^**^ | 1.00 |  |  |  |  |  |  |  |  |  |  |  |
| VI. Shyness | .254 | .028 | .676^**^ | .275 | .46 | 1.00 |  |  |  |  |  |  |  |  |  |  |
| VII. Fatigue | -.142 | -.126 | .279 | .002 | .097 | .352 | 1.00 |  |  |  |  |  |  |  |  |  |
| VIII. Serenity | .077 | .012 | -.275 | .297 | .191 | -.101 | .078 | 1.00 |  |  |  |  |  |  |  |  |
| IX. Surprise | -.121 | .081 | .424 | .575^*^ | .542^*^ | .327 | .07 | .058 | 1.00 |  |  |  |  |  |  |  |
| X. Fear | -.020 | .082 | .836^**^ | .330 | .380 | .606^**^ | .418 | -.284 | .345 | 1.00 |  |  |  |  |  |  |
| XI. Hostility | -.108 | -.199 | .732^**^ | .222 | .165 | .507^*^ | -.093 | -.244 | .435 | .353 | 1.00 |  |  |  |  |  |
| XII. Guilt | -.052 | .089 | .864^**^ | .148 | .068 | .449 | .144 | -.324 | .473 | .608^**^ | .766^**^ | 1.00 |  |  |  |  |
| XIII. Sadness | -.030 | -.186 | .648^**^ | .088 | .215 | .678^**^ | .544^*^ | -.189 | .176 | .673^**^ | .439 | .363 | 1.00 |  |  |  |
| XIV. Joviality | -.029 | -.044 | .252 | .858^**^ | .596^**^ | .069 | -.002 | .363 | .284 | .249 | .068 | .066 | -.057 | 1.00 |  |  |
| XV. Assurance | -.279 | -.055 | .158 | .771^**^ | .518^*^ | .077 | -.031 | .391 | .364 | .085 | .190 | .077 | .082 | .694^**^ | 1.00 |  |
| XVI. Control | .324 | .506^*^ | -.232 | .063 | .261 | .019 | -.383 | .147 | -.079 | -.084 | -.238 | -.235 | -.197 | -.026 | -.006 | 1.00 |
